# The Impact of Social Factors on Job Crafting: A Meta-Analysis and Review

**DOI:** 10.3390/ijerph17218016

**Published:** 2020-10-30

**Authors:** Huatian Wang, Peikai Li, Shi Chen

**Affiliations:** 1 Industrial Engineering and Innovation Science, Eindhoven University of Technology, 5600 MB Eindhoven, The Netherlands; h.wang4@tue.nl (H.W.); s.chen2@tue.nl (S.C.); 2 Social, Health and Organizational Psychology, Utrecht University, 3584 CS Utrecht, The Netherlands

**Keywords:** job crafting, meta-analysis, leadership, social factors

## Abstract

Despite the considerable focus on job characteristics and individual differences in job crafting research, the influence of social factors on job crafting has not been well-acknowledged. Based on social interaction and job crafting literature, this meta-analysis estimates the associations between social factors (i.e., organizational insiders and outsiders) and job crafting, and how these social factors contribute to employee outcomes through their job crafting. Based on a sample of 51 empirical studies that included 54 independent samples (N = 17,863), we found that social factors of positive leadership styles (e.g., empowering and transformational) and coworker support were positively related to employee job crafting. Moreover, leadership showed a stronger correlation with employee job crafting than coworker support and Leader-Member-Exchange (LMX). Further, our study showed that employee job crafting positively mediates the relationships between social factors and work outcomes (e.g., job performance and well-being). Our study contributes to job crafting literature by integrating social factors into the job crafting model and demonstrating that the social context of work (in particular organizational insiders) plays a crucial role in shaping employees’ job crafting behavior. We also emphasize the critical role that job crafting plays in transmitting valuable social resources into improved work outcomes. Building on our results, we provide future direction for job crafting research and discuss how our results can imply practice in terms of job crafting training.

## 1. Introduction

Job crafting, referring to actions employees take to change the task, relation, or cognitive boundaries of a job [1,2], has been a focal research topic in job design literature since the early 2000s [3]. Employees’ initiated job crafting behavior (e.g., seeking resources and seeking challenges) has been positively linked to employee health, job attitude (e.g., job satisfaction), well-being (e.g., work engagement), and performance (for meta-analytic reviews, see [4,5]). It also brings substantial benefits for organizations, such as a higher level of group and organizational performance [6]. Accordingly, increasing research has investigated various ways to stimulate employees’ job crafting behavior. In this respect, cumulative evidence has shown that job characteristics and personal traits are important factors that influence employee job crafting (for reviews, see [4,7]). For example, proactive personality [8], self-efficacy [9], regulatory focus [5], job autonomy [4], and job resources [10] were positively related to employee job crafting.

While prior studies have provided valuable insights into how personal traits/abilities and job characteristics linked to employee job crafting [4,7], a recent and growing number of studies examined how social factors influence employee job crafting (e.g., [2,7,9]). Social elements of work may play a crucial role in influencing employees’ behavior [11]. It represents social connections that employees access in work domains (e.g., leaders, colleagues, customers, clients, and patients) and non-work domains (e.g., families and friends) [12]. The interactive societal environment encompasses opportunities and resources that are vital to foster individual self-growth, career success, and need satisfaction [13,14]. Understanding how employees learn from their social connections may be as important as understanding who they are and what their jobs look like. While meta-analyses and review articles already exist in the area of job crafting (i.e., [4,5,7,15]), a comprehensive review of social factors and job crafting is still absent. To our knowledge, Tims and Parker (2020) [16] took such an endeavor but their attention was limited to how colleagues respond to the crafter’s behavior. Likewise, in a review article of Zhang and Parker (2019) [7], the social factors only include leaderships (e.g., transformational and empowering leadership). Hence, it is concluded that these studies do not give a full overview of the impact of social factors on job crafting. More importantly, not all empirical studies find favorable results of social factors on job crafting [7,16]. For example, while some studies showed a positive relationship between transformational leadership and promotion-focused job crafting [17,18,19], others showed a nonsignificant relationship [19,20]. Similarly, Loi et al. (2019) [21] indicated a positive relationship between Leader-Member-Exchange (LMX) and job crafting, whereas Radstaak and Hennes (2017) [22] found a negative correlation with increasing structural resources. Overall, the effect of social factors on job crafting looks quite complex and uncertain. We have limited knowledge about the extent to which social factor has a stronger and significant impact on employee job crafting. Therefore, a meta-analysis will help clarify the relationship between social factors and job crafting and estimate the extent to which social factor is more important to employee job crafting.

**Figure 1:**
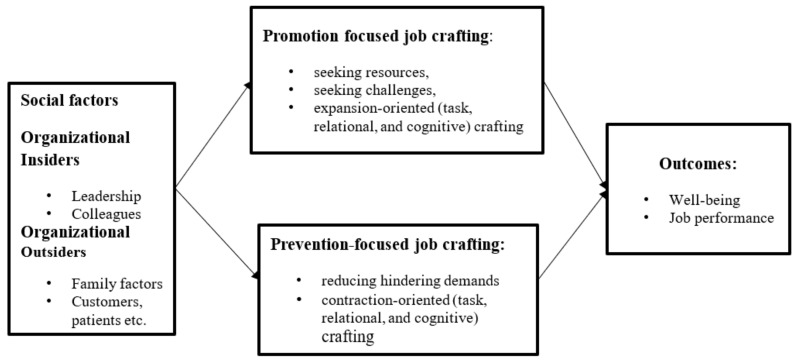
Hypothesized model.

The main purpose of this study is to provide a meta-analytic review of the associations between social factors and employee job crafting and uncover how job crafting acts as a mediator linking social factors and work outcomes. To organize this effort, we integrate extant research into a conceptual model that extends previous reviews and meta-analysis [4,7] by grouping social factors into organizational insiders and organizational outsiders (see Figure 1). Meanwhile, we considered job crafting into two ways: promotion and prevention-focused job crafting; and the different forms of job crafting defined by Tims et al. [23] (e.g., seeking resources, seeking challenges and reducing demands).

This study contributes to the literature in three respects. First, we contribute to the job crafting literature by analyzing the relationship between different social-elements antecedents (e.g., leadership and coworkers) and job crafting. Using a meta-analytic approach, we provide meta-analytic evidence about how different social factors (i.e., different leadership styles and coworker factors) related to different forms of job crafting, which advances our knowledge about the antecedents of job crafting. Second, our study contributes to job crafting literature by underlying that job crafting plays an important mediating role in the relationship between social factors and work outcomes. That is, job crafting acts as an effective self-initiated strategy that may successfully transform favorable social factors into improved work performance and well-being. We extend the work of Lichtenthaler and Fischbach [5] and uncover the mediating role of promotion-focused job crafting in the interpersonal context. In doing so, we provide a finer-grained understanding of how social factors related to employee outcomes through job crafting. Finally, as research on social factors and job crafting is in a relatively early stage, our review provides a unique opportunity to identify gaps in the literature with regards to theory and methodology to guide future research.

## 2. Theory and Hypothesis

### 2.1. Job Crafting Conceptualizations

Historically, job crafting was first introduced by Wrzesniewski and Dutton [1]. The authors defined job crafting as employee self-initiated physical and cognitive changes in tangible work role boundaries and intangible work role perceptions. Three types of job crafting were included—task crafting (i.e., changing the tasks individuals perform at work), relational crafting (i.e., changing the social characteristics of the job), and cognitive crafting (i.e., changing the way individuals think about their jobs). The role-based job crafting approach posits that job crafting motives are rooted in employees’ needs to gain control, a positive self-image, and social relatedness at work [24]. It can satisfy individual basic needs, contribute to increased meaningfulness at work, and gain related benefits of work meaningfulness [5].

Subsequently, Tims and Bakker [25] defined job crafting as the self-initiated changes employees make to adjust their job resources and job demands. This conceptualization of job crafting is situated in the broader framework of the job demands-resources (JD-R) model [26]. Three types of job crafting were identified– seeking resources (e.g., ask for feedback), seeking challenges (e.g., ask for more responsibilities), and reducing hindering demands (e.g., making work less mentally intense). Based on the JD-R model, resource-based job crafting motives are rooted in employees’ need to align their levels of job resources and job demands with their own abilities and preferences [4,27]. Job crafting promotes a person-environment fit, work engagement, and reduces job strain and burnout [26].

Although JD-R job crafting stimulates numerous studies, there are still two limitations. An important limitation is that Tims and Bakker’s approach [23], to a large extent, removed cognitive crafting, whereas the significance of cognitive crafting was to be proved by previous studies [7]. The other limitation of the JD-R crafting is that it may ignore employees’ motivation of job crafting. Therefore, Lichtenthaler and Fischbach [5] took a regulatory focus perspective to refine and integrate previous two job crafting conceptualizations in order to grasp the essentials of job crafting. Regulatory focus theory is a common theory to explain employees’ motivation and proactive changes at work [25]. It posits that individual behaviors tend to involve two self-regulatory processes—promotion-focused process and prevention-focused process. The former one emphasizes that people are motivated to approach positive end-states where their needs are satisfied, whereas the latter one underlines that people are motivated to avoid negative end-states where their needs are not satisfied [28,29]. Accordingly, two types of job crafting were proposed—promotion-focused job crafting and prevention-focused job crafting. Promotion-focused job crafting refers to the self-initiated changes employees make to expand and approach resources and challenges with a promotion focus; while prevention-focused job crafting refers to those self-initiated changes employees make to avoid and contract resources losses with a prevention focus [5]. Examples of promotion-focused job crafting are seeking resources, seeking challenges, expansion-oriented task, relational, and cognitive crafting. Prevention-focused job crafting includes reducing hindering demands, contraction-oriented task, relational, and cognitive crafting [23,30].

In sum, regulatory-focused job crafting introduces motivation into the job crafting field, which can help us to capture a more comprehensive understanding of different motivational processes (e.g., promotion-focus and prevention-focus) behind job crafting behaviors. Drawn on regulatory-focused job crafting, we can easier account for the beneficial and detrimental effects of job crafting [5]. In addition, regulatory-focused job crafting integrates role-based job crafting and JD-R job crafting, which helps us interpret job crafting in a broader and deeper perspective. Therefore, we will use the forms of promotion-focused job crafting and prevention-focused job crafting to probe their antecedents and mediating roles.

Notably, since the majority of this field used the JD-R job crafting, which is in line with Rudolph et al. (2017) [31], in this meta-analysis, we also tested how social factors related to employees’ specific job crafting behaviors (i.e., increasing structural resources, increasing social resources, increasing challenging demands, and reducing hindering demands) as conceptualized by Tims et al. (2013) [23].

### 2.2. Antecedents of Job Crafting: Social Factors

Prior literature on the investigation of job crafting has typically followed one of three main factors—individual difference (e.g., personality in the framework of Big Five model), job characteristics, or demographics (e.g., age, tenure, and work hours)—to understand the factors that associated with job crafting [4,7]. For example, research found that individual personality, self-efficacy, self-competence, and demographics of age and gender may influence job crafting [4,32,33]. Job characteristics of job autonomy, job enlargement, task significance, and task identity are positively related to employee job crafting [34,35].

Although the job crafting literature has well recognized the associations between individual factors and job characteristics factors and employee job crafting, a growing number of studies investigate the impact of social factors on job crafting [7]. For example, scholars start to realize the important role of leadership and colleagues on individual job crafting [7,16,36]. These positive social interactions include resource exchange and share, and in turn, motivate employees to adjust their job boundaries [1,2]. In the current study, by integrating the socialization literature [37], we argue that two types of social factors will influence employee job crafting: (a) organizational insiders, including leaders [36], colleagues [16]. We emphasize the inherently “interpersonal” nature of organizational insiders as sources of social resources (e.g., information sources; c.f., [37,38]) and (b) organizational outsiders, including customers, patients, clients, and family members, may have a direct or indirect effect on employee’s task or employee themselves. We borrow this framework because it more fits the interpersonal context of employee job crafting. When employees intend to craft their jobs, social interactions such as interactions with leaders, colleagues, clients, or even families will play an important role. Below we explain how these social factors impact job crafting behaviors.

#### 2.2.1. Interacting with Organizational Insiders

Leadership. The leader is one of the most important organizational-insider social factors that influence employee job crafting. Several resources-based theories can explain why social factors influence employee job crafting. According to the JD-R theory, leaders tend to have more valuable job resources that can offer to employees [39,40]. In particular, leaders promote individuals job crafting by providing personal resources and social support to employees and designing resourceful jobs with urgency to craft [36]. This is because supportive leaders (e.g., servant leadership) are usually seen as the valuable interpersonal resources for employees [38,39]. Employees can learn and imitate from their leaders’ good actions and thoughts [36,37]. Leaders are able to offer employees with job resources such as autonomy for jobs, empowerment to employees, additional opportunities, and valuable feedback [41,42]. These job resources enable employees to feel engaged and experience positive affect during the work, thereby promoting their motivation to modify tangible work role boundaries and intangible work role perceptions [4,7]. Therefore, we argue that favorable leadership styles will provide employees with social support and interpersonal resources, which prompts their job crafting actions. Empirical studies found that employee-oriented leadership [43], transformational leadership [19], empowering leadership [41,44] and servant leadership [39,45] were positively related to employee promotion-focused job crafting. Those leaders provide autonomy to employees and encourage employees to pursue long-term goals, thereby promoting employees’ motivation to craft their jobs. In addition, those leaders can influence job crafting via promoting employee’s organizational identification and building a trusting, open, and supportive work climate [36].

However, not all leaders constantly provide resources to employees. Some leaders bring hindering demands/stressors to employees (e.g., destructive or abusive leadership) [46,47]. When employees perceive a limited scope for action, unilateral decisions about a job’s tasks and goals, or an attitude from the leader that could be perceived as incorrect, they will most likely to adjust the job to decrease hindering job demands/stressors (a type of prevention-focused job crafting). Esteves and Lopes [20] found that directive leadership was positively related to prevention-focused job crafting. Tuan [48] found a negative association between authoritarian leadership and promotion-focused job crafting. Therefore, we argue that some favorable leaderships such as transformational, empowering, and servant leadership may contribute to employee promotion-focused job crafting, whereas some destructive leadership, such as authoritarian and abusive leadership may lead to employee prevention-focused job crafting.

Colleagues. Colleagues are another important aspect of organizational-insider social factors. Tims and Parker [16] recognized that increasing employees may work in a group or team context. Therefore, coworkers play an increasingly critical role in employee job crafting [49]. According to the JD-R theory [26], colleagues provide employees with useful job resources as well. Colleagues are often taken as a form of social support which provides timely assistance, valuable feedback, and unique perspectives [12,50]. Employees are motivated to adjust their work procedures and tasks if they receive timely help and useful feedback towards current hurdles. Hence, employees may feel engaged and motivated to enact their jobs when perceiving a higher level of interpersonal resources and social support, and in turn, proactively adjust their jobs [5,30]. Prior studies showed that colleagues’ helping behaviors and support were positively related to focal employee promotion-focused job crafting [51]. Similarly, excellent colleagues can function as role-senders, who communicate expectations about tasks [52]. Individuals are willing to imitate good behaviors and learn new knowledge from others in order to promote their own behavior and performance [51]. By contrast, if employees encounter conflicts with colleagues, this interaction will incur negative effects. Employees may feel stressful and identify this event as a hindering demand/stressor [20,26,46]. As a result, they may take an avoidance strategy—a form of prevention-focused job crafting. In summary, we argue that colleagues are another form of social factors in workplaces that may shape employee job crafting.

#### 2.2.2. Interacting with Organizational Outsiders

Clients/Customers. Besides social factors, such as leaders and colleagues in the work domain, employees’ serve objects may also play an important role in employee job crafting. For instance, for the service industry (e.g., education, tourism, and medical), customers and clients are also important social factors. Loi et al. [21] found that customer participation positively related to cognitive and relational job crafting. The reason is that customer participation contributes contextual resources and support to service employees by means of taking up part of employees’ work tasks and providing employees with new knowledge and information [53]. When employees perceive more participation from their customers, they are more likely to conduct task crafting such as altering the number and scope of their jobs or expanding to perform different tasks. Auh et al. [54] found that clients’ expertise and advice add value to service delivery in the financial planning context by means of a joint decision-making process. It is expected that these positive processes facilitate how employees view their jobs, to develop personal resources such as becoming more innovative, and enable employees to gain a positive sense of self and meaningfulness [21]. By contrast, if employees encounter increasing needs from clients or customers, these customized needs may increase employees’ pressure and workloads, and in turn drain employees’ energy [26]. As a result, employees may be very likely to take an avoidance strategy such as a prevention-focused job crafting action.

Families. In addition to clients and customers, social factors in the non-work domain may also influence employee job crafting. Research suggests that resources have spill-over effects across the work domain to non-work domain, and vice versa [55]. Westman [55] underlines that experiences, emotions and resources can be transferred across social and organizational contexts. For example, positive emotions and experiences outside work may be transferred to the work domain. Crossover of resources acts as a mechanism of resource exchange within resource caravans [56]. Such an exchange triggers the accumulation of resources in a positive way [57]. To achieve one’s goals, individuals are motivated to self-expand one resource to increase other resources [58].

We thus argue that organizational outsiders such as family-based social factors may be an important antecedent of job crafting. Family-based social factors mainly include positive aspects such as family support and negative aspects such as family-work conflicts. Based on a resource-based view, the former may provide family instrumental and emotional support [59]. It is argued that resources (e.g., social support) gained from the non-work domain can promote individual performance and affect in the work domain [60]. For example, emotional support from families lead individuals to feel loved, cared for, and valued [61], which can foster positive affect in the family to transfer to the individual’s functioning at work. As a result, those employees who feel supported would keep motivated and engaged to enact their jobs and present proactivity in adjusting work role boundaries and perceptions. Research suggests that external support and a supportive environment are positively related to job crafting [59,62]. Therefore, we argue that positive family factors such as family support may lead to promotion-focused job crafting.

However, the negative family factors (i.e., family-work conflicts) may lead to negative consequences such as resources loss and negative affect [63,64]. Research has shown that family-work conflicts lead to negative affect and resource loss, and these negative events could further impact individual affect and behaviors in the work domain [59,64,65]. As a result, employees may carry the negative affect and feel stressed during work time and perceive multiple demands of work and family roles. Hobfoll et al. [55] posit that individuals are motivated to protect their current resources and avoid resources loss in order to cope with threats and stress. Hence, employees may adopt an avoidance strategy (e.g., reducing job demands) to decrease exhaustion during the work. That is, family-work conflicts may trigger employees to reduce work demands to meet family demands. We argue that family-work conflicts may lead to prevention-focused job crafting.

Taking together, we argue that social factors including interacting with leaders, colleagues, clients, and families may significantly influence employees’ motivation to enact and craft their jobs. Therefore, we hypothesize:

**Hypothesis** **1** **(H1).**  *Social factors (leadership, coworkers, and family factors) are related to employee job crafting.*

Specifically:

**Hypothesis** **1** **(H1a).**  *Favorable social factors (e.g., supportive leaders, colleagues, clients, and families) will be positively related to promotion-focused job crafting.*

**Hypothesis** **1** **(H1b).**  *Favorable social factors are negatively related to prevention-focused job crafting.*

**Hypothesis** **1** **(H1c).**  *Destructive social factors (e.g., abusive leaders and conflicts with colleague, clients, or families) will be negatively related to promotion-focused job crafting.*

**Hypothesis** **1** **(H1d).**  *Destructive social factors will be positively related to prevention-focused job crafting.*

### 2.3. Social Factors, Job Crafting, and Work Outcomes

In the previous section, we argued that social factors influence employee job crafting behaviors. However, another important question is whether job crafting can serve as a mediating tool linking social factors and work outcomes.

Job crafting literature indicates that promotion-focused job crafting is positively related to various work outcomes [4,7]. Based on the JD-R theory, promotion-focused job crafting is able to effectively mobilize resources, set challenging goals, and behave innovatively to facilitate work performance [15,25]; meanwhile, via promotion-focused job crafting, employees can effectively cope with threats and stress and thereby obtain and maintain a higher level of positive attitudes and occupational well-being [6,66]. Hence, promotion-focused job crafting can not only facilitate task completion (work performance), but also make employees feel good at work (well-being). Empirical studies have shown that promotion-focused job crafting is positively related to creativity [67], subjective well-being [68], positive affect [69], career competence [70], and career satisfaction and commitment [41].

Accordingly, we argue that promotion-focused job crafting would be a salient mediating mechanism linking social factors and beneficial work outcomes. In this study, we categorize work outcomes into two aspects: employee performance and well-being, which are consistent with the general model of proactive behavior by Bindl and Parker [33]. These two are important indicators to measure employee work outcomes because they emphasize what employees achieve and how employees feel during the work. Prior studies showed that employee performance usually comprises task performance, creative performance, and extra-role performance; whereas employee well-being usually comprises job satisfaction, turnover intention, and thriving [4,7]. Following our theoretical reasoning so far, it is expected that social factors are positively related to job crafting, and subsequently, job crafting (particularly, promotion-focused job crafting) is positively related to work performance and well-being.

For example, some favorable leaderships such as empowering, transformational, servant leadership can effectively promote employee work performance and well-being through promotion-focused job crafting. This may be because favorable leadership allows employees with more job resources such as job autonomy and support [35,39,40]. With increased job autonomy and support, employees are motivated to craft their work role boundaries and perceptions, and in turn, gain a higher level of task performance and positive affect at work [4,7]. Likewise, colleague and family support provides employees with additional social resources. These additional social resources are often seen as social capitals, referring to those actual and potential resources embedded within, available through, and derived from the network of relationships [12]. With increased social capitals, employees have more confidence and motivation to seek resources and avoid resources loss at work, which can influence their work performance and affect at work [16,71]. As a result, promotion-focused job crafting may serve as a significant mediating role. For example, some studies found that promotion-focused job crafting can successfully transmit the benefits of leaderships into improved innovative work behavior and citizenship behaviors towards coworkers and customers [17,39]. Besides, Guan and Frenkel [72] uncovered that job crafting effectively mediated the positive human resource management (HRM)–performance relationship. Zito et al. [73] confirmed the positive association between job autonomy and job satisfaction through promotion-focused job crafting. Therefore, it is expected that promotion-focused job crafting is an effective mediating process by which social factors are successfully transformed into increased work performance and well-being.

On the contrary, those unfavorable social factors such as destructive leadership and clients, family-work conflicts tend to result in prevention-focused job crafting. The reason is that employees likely perceive such social factors as hindering demands and thus possibly adopt an avoidance strategy (e.g., prevention-focused job crafting) at work [20]. Research further suggests that prevention-focused job crafting (e.g., reducing hindering demands) may have negative or nonsignificant effects on employee outcomes [23,30]. This is because hindering job demands seem to be given job characteristics which employees cannot change easily by themselves [23]. This means that prevention-focused job crafting such as reducing hindering demands is likely to be unsuccessful and does not produce favorable changes in tangible work role boundaries. Moreover, dealing with hindering job demands leads to work-withdrawal behaviors, which reduces work engagement [5]. As a result, employee job crafting with prevention focus may involve a less motivational process and can hardly transform these hindering social factors into improved work performance and well-being. Therefore, we expect that unfavorable social factors link to prevention-focused job crafting, and in turn, have detrimental effects on work outcomes. We hypothesize:

**Hypothesis** **2** **(H2).**  *Promotion-focused job crafting mediates the positive relationship between favorable social factors and (a) work performance/(b) well-being.*

**Hypothesis** **3** **(H3).**  *Prevention-focused job crafting mediates the negative relationship between unfavorable social factors and (a) work performance/(b) well-being.*

## 3. Methods

### 3.1. Literature Search

To identify as many published and unpublished studies as possible, following the “best practices of literature search” in systematic reviews and meta-analyses [31,74,75], we conducted three sets of literature searches. First, online databases search. Relevant studies were identified through searching for eight databases: Web of Science, PsycINFO, Google Scholar, Journal Storage (JSTOR), ScienceDirect, EBSCO, Emerald, and ProQuest. We used AND OR Boolean search operators to define our search strings (for an example in Web of Science, see Appendix A). In particular, our keywords consist of two components: job crafting and social factors. For searching job crafting, we used keywords such as “job crafting” or “crafting”; for searching social factors, we used keywords such as “leadership”, “leader”, “manager”, “colleague”, “coworker”, “work-family”, or “work-home”. All of the searched literature was updated until June 2020. Second, to increase the accuracy and quality of literature search, as suggested by “best practices of literature search”, we also conducted a manual search from 12 highly relevant journals (i.e., *Journal of Applied Psychology, Journal of Management, Journal of Organizational Behavior, The Leadership Quarterly, Journal of Occupational Health Psychology, European Journal of Work, and Organizational Psychology, Personnel Psychology, Journal of Occupational and Organizational Psychology, Journal of Business Psychology, Human Relations, Academy of Management Journal*, and *Journal of Vocational Behavior*). These journals are recognized as high-quality journals in work and organizational psychology field (see latest Journal Citation Reports, www.webofknowledge.com). These selected journals are also manually searched by previous job crafting meta-analysis [5,7,31]. Finally, we conducted backward search by recognizing three published meta-analytic articles concerning job crafting (i.e., [4,5,7] and checked their references list. In total, we identified 2059 potential articles for title and abstract screening after excluding duplicates.

### 3.2. Inclusion Criteria and Study Coding

**Figure A1:**
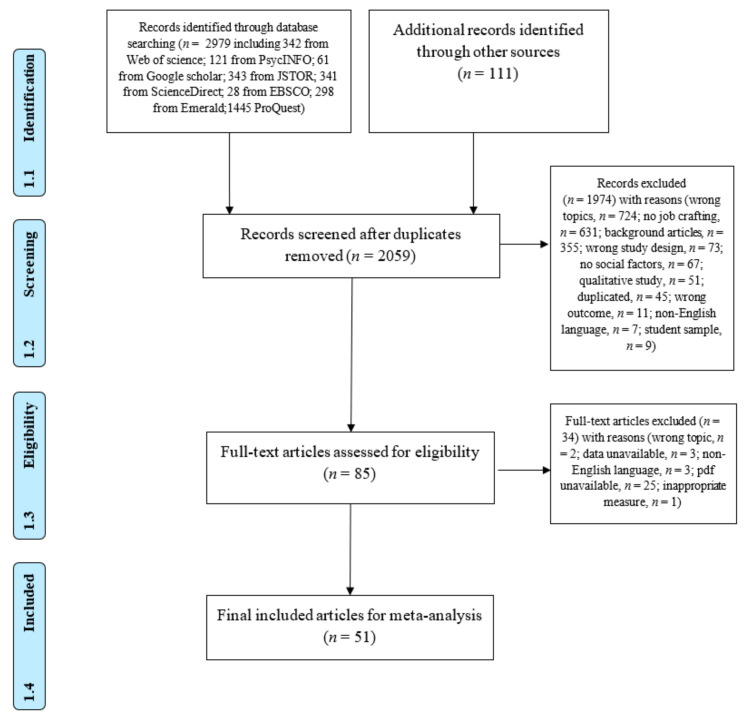
The flow diagram of literature searching process. Note: Figure is adapted from Moher, Liberati, Tetzlaff, Altman, The PRISMA Group, 2009.

We included studies in the meta-analysis using the following criteria: first, studies had to be a published quantitative study. Qualitative, review, or case studies were excluded. Second, studies had to include measures of both job crafting and social factors. Specifically, the measure of job crafting can be either (a) an overall job crafting, or (b) four specific job crafting dimensions developed by Tims et al. [27] such as increasing social resources, increasing structural resources, increasing challenging demands, and decreasing hindering job demands, or (c) three specific job dimensions based on seminal work of Wrzesniewski and Dutton [1], such as task crafting, cognitive crafting, and relational crafting, or (d) two specific job dimensions based on Lichtenthaler and Fischbach [5], such as promotion/expansion-oriented job crafting and prevention/contraction-oriented job crafting. The measures of social factors can be either (a) different types of leadership such as transformational, empowering, servant, authentic, charismatic, and transactional leadership, or leadership related concepts such as leader-member exchange (LMX); (b) colleagues, coworkers, such as colleagues’ job crafting behaviors and coworkers’ support, or (c) family factors such as work-family interference. Third, the correlations between job crafting (or specific dimensions of job crafting) and social factors had to be reported in the studies. Fourth, when a study used two or more independent samples (e.g., [76]), samples were coded separately. When the same sample was used in more than one article (e.g., [77,78], we made sure to include the same relationships only once. These inclusion criteria yielded in a final set of 51 studies representing 54 independent samples with 17,863 employees (see Appendix B, Figure A1 provides a flow chart of our searching process).

All 51 studies were coded by two authors independently and the coding was compared in cases of disagreement. Interrater agreement was 94% across the study variables, indicating substantial agreement. Disagreements about the inclusion of a study or specific coding were discussed until a consensus was achieved. For each study, we coded the sample size, the correlations between social factors and job crafting, the correlations between social factors and outcome variables, the correlations between job crafting and outcome variables. These data were further checked by another author to reduce potential coding error. Moreover, we coded additional information including study design, theories, employees’ demographic backgrounds such as age, tenure, gender, nationality, and occupation, and main findings. Appendix C provides a table that lists all the included studies.

### 3.3. Data Analysis Strategy

Random-effects meta-analytic procedures [79] were conducted in R program by using the R ‘metafor’ package [80]. The random effect models (Hunter and Schmidt, 2004) allow for the possibility that the population parameter values differ across studies in our sample because they come from different subpopulations (e.g., different regions or countries) [79]. To provide accurate estimates, the weighted mean correlations and their variances were corrected for sampling error [79]. When a study reported effect sizes for multiple independent samples, all of the relevant correlations were included as separate effect sizes. As some studies reported correlations of specific job crafting (e.g., increase social resources, increase challenge demands, and increase job resources) and social factors, these data are nested in the same sample. When calculating the overall effect sizes between social factors and job crafting, we used the three-level meta-analysis to calculate the pooled effect sizes as researchers suggested it is a better way to address the dependency of effect sizes issue [81,82]. We used two indexes to assess between-study heterogeneity: Q-test and I^2^ (i.e., an index of heterogeneity computed as the percentage of variability in effects sizes that are due to true differences among the studies) [83]. A significant Q test value indicates that the studies are more heterogonous as one would normally expect. I^2^ index suggests the percentage of variation across studies due to heterogeneity [83]. Correlations were considered as statistically significant when the 95% confidence interval (also a measure of variability distribution of correlations across studies) did not include zero.

In addition, for sensitivity analysis, we (a) corrected the correlations for artifact distributions of measurement error using Cronbach’s alpha (α) coefficient of internal consistency (i.e., for social factors and job crafting); For studies do not report alpha, we used a mean Cronbach’s alpha from other studies in this meta-analysis; (b) used outlier analysis to correct for the potential impact of extreme effect sizes; (c) and the trim-and-fill procedure was used to correct for potential publication bias.

To examine the mediating effect, we conducted the meta-analytic structural equation modeling approach (MASEM) [80]. A two-stage structural equation modeling (TSSEM) approach was employed to test how social factors influence outcomes through job crafting [84]. And the R metaSEM package was used to perform our analyses [80,84]. In the first stage, we combined the relevant effect sizes into matrices to calculate a pooled correlation matrix; next, we estimated the mediation effect by fitting a structural equation model to the pooled meta-analytic correlation matrix. We requested a 95% confidence interval around the indirect effect and considered as statistically significant when it did not include zero.

## 4. Results

### 4.1. Social Factors and Job Crafting

Due to dependency of effect sizes in our study (i.e., some studies reported more than one effect sizes of different job crafting behaviors), we used the three-level meta-analysis approach to test the overall effect of social factors on job crafting. The results indicated that overall social factors are positively related to promotion-focused job crafting (k = 68, ρ = 0.372). About 5.8% of the overall variance can be attributed to level 1 (i.e., sampling variance), 73.8% to level 2 (variance between effect sizes extracted from the same study), and as much as 20.4% to level 3 (variance between studies). And the overall three-level model compared to the reduced two-level model does indeed have a better fit, with the Akaike Information Criterion (AIC) and the Bayesian Information Criterion (BIC) being lower for this model (likelihood-ratio test = 45.56, *p* < 0.001). The difference is significant, suggesting we should include this level into our analysis.

Hypothesis 1 states that social factors are related to job crafting. The three-level meta-analysis results showed that most of the variance of effect sizes are caused by the Level 2 variance (i.e., different types of social factors and job crafting behaviors), thus we investigated how specific social factors related to specific job crafting behaviors. Table 1 reports the relationships between social factors and job crafting when considering job crafting as promotion-focused job crafting and prevention-focused job crafting. Table 2 reports the relationships between social factors and job crafting when considering job crafting as increasing structural resources, increasing social resources, and increasing challenges demands. For this analysis, we only included one effect size from each sample. Meta-analyses results in Table 1 demonstrated that social factors were positively related to employee promotion-focused job crafting (k = 32, ρ = 0.361, CI = (0.292, 0.426)). The subgroup analysis showed that social factors of coworker support (k = 3, ρ = 0.237), leadership (k = 22, ρ = 0.400), and LMX (k = 7, ρ = 0.277) were positively associated with promotion-focused job crafting. And leadership showed a stronger mean-corrected correlation with employee job crafting than coworker and LMX (t = 4.90, *p* = 0.026), but there is no significant difference between coworker and LMX (t = 0.207, *p* = 0.648). When we focused on the associations between social factors and specific job crafting, which showed that social factors were positively related to promotion-focused job crafting of increasing structural resources (k = 6, ρ = 0.178, CI = (0.058, 0.293)), increasing social resources (k = 10, ρ = 0.332, CI = (0.246, 0.414)), and increasing challenge demands (k = 11, ρ = 0.210, CI = (0.138, 0.277)) (see Table 2). Hypothesis 1a was supported.

Unexpectedly, we found insignificant effect of social factors on prevention-oriented job crafting (k = 9, ρ = 0.022, CI = (–0.091, 0.134)) (see Table 1). Hypothesis 1c was not supported.

Due to the lack of sample sizes on destructive social factors, such as destructive leaders, conflicts with clients and families, Hypothesis 1b and 1d were not tested.

To present more detailed results of specific social factors on job crafting, below we report how specific social factor influences employee job crafting behaviors.

#### 4.1.1. Leadership and Job Crafting

We found that leadership was positively related to employee promotion-focused job crafting behavior (k = 22, ρ = 0. 400, CI = (0.314, 0.480)). Specifically, leadership styles of empowering (k = 7, ρ = 0.338), transformational (k = 5, ρ = 0.270), charismatic (k = 3, ρ = 0.160), servant (k = 3, ρ = 0.686), and transactional (k = 3, ρ = 0.236) are positively related to promotion-focused job crafting. When we consider how leaderships are related to specific job crafting behaviors. We found that empowering leadership and transformational leadership are two salient social factors. In particular, empowering leadership was positively related to increasing social resources (k = 4, ρ = 0.368, CI = (0.181, 0.530)) and increasing challenge demands (k = 4, ρ = 0.305, CI = (0.174, 0.426)), respectively. Transformational leadership was positively related to increasing social resources (k = 3, ρ = 0.367, CI = (0.196, 0.517)), increasing structural resources (k = 3, ρ = 0.260, CI = (0.078, 0.425)), and increasing challenges demands (k = 3, ρ = 0.234, CI = (0.165, 0.300)).

In addition, some of our included studies tested the effect of team-level leadership on job crafting (which were not included in the meta-analysis to calculate the pooled effect size). For instance, team level servant leadership (Luu et al., 2019; Tuan et al., 2020), charismatic (Luu et al., 2019) are positively related to job crafting. Besides, in our reviewed articles we also found that some destructive leadership styles have a negative effect on employee job crafting. For instance, abusive supervision (*r* = −0.24, Luu et al., 2019), leader’s need for structure (*r* = −0.14, Solberg and Wong, 2016), and paternalistic leadership/ authoritarianism (*r* = −0.26, Tuan, 2018) are negatively related to employee job crafting. These are in line with our Hypothesis 1b.

#### 4.1.2. Coworkers and Job Crafting

We found that coworker emotional and instrumental social support are positively related to employee promotion-focused job crafting (k = 3, ρ = 0.237, CI = (0.108, 0.358)) (see Table 2). In addition, colleagues’ job crafting also influences employee job crafting behaviors. For instance, Bakker et al. [85] showed a reciprocal relationship between dyad members’ job crafting behaviors—each of the actor’s job crafting behaviors was positively related to the partner’s job crafting behaviors. Similarly, Demerouti and Peeters [51] found the transmission of both job crafting dimensions among colleagues. Similar cross-over effect was reported by Peeters, Arts, and Demerouti [71].

In our reviewed articles, we only found one article regarding the factor of clients/customers (r = 0.38, Loi et al., 2029 [21]). Due to such little sample size, we did not include this article in our meta-analysis. Moreover, we found that only few studies focused on the associations between family factors and job crafting. For instance, we found that work-family conflict encourages or discourages job crafting by moderating the relationship between tendencies toward workaholism and expansion and contraction-oriented job crafting [86]. Job crafting is positively related to work-family conflict [73], and work-to-family enrichment [21,42,87]. However, the latter three studies treated family factors as outcomes, thus did not focus on how family factors influence job crafting.

In summary, we found that positive social factors especially organizational insiders were positively related to promotion-focused job crafting. Thus, Hypothesis 1a was supported. Whereas the results between social factors and prevention-focused job crafting were insignificant, Hypothesis 1c was not supported. We do not have enough samples to test how negative social factors related to promotion and prevention focused job crafting, thus, our Hypotheses 1b, and d were not tested.

### 4.2. Sensitivity Analysis

#### 4.2.1. Correction for Measurement Error

We used Cronbach’s alpha coefficient of internal consistency to correct correlations for artifact distributions of measurement error for perceptions of social factors and employee job crafting. The results were presented in Table 1 and Table 2 show that the corrected effect sizes were higher than sampling-weighted effect sizes. However, it does not influence our conclusions.

#### 4.2.2. Outlier Analysis

Our sensitivity analyses showed that after removing outliers (i.e., the study’s confidence interval does not overlap with the confidence interval of the pooled effect, Harrer et al., 2019) [88], most results did not differ much from the overall meta-results (see Table 1 and Table 2). We found positive correlations between social factors and promotion-focused job crafting (with 1 study removed, k = 21, ρ = 0.341), which is lower than the original effect sizes (ρ = 0.361). Similarly, leadership is positively related to promotion-focused job crafting (with 5 studies removed, k = 17, ρ = 0.385). This showed that due to some outliers exist, for some of our tested correlations the original pooled effect sizes might be overestimated.

#### 4.2.3. Publication Bias

To test potential publication bias, we used the trim-and-fill procedures. A test of the null hypothesis that the number of missing studies (on the chosen side) is zero was retained for the associations between promotion-focused job crafting and overall social factor, coworker support, overall leadership. For the associations between servant leadership (with two studies added k = 5, ρ = 0.670), LMX (with 3 studies added k = 10, ρ = 0.174) and promotion focused job crafting were lower than original pooled effect size. This suggests due to publication bias, some of our initial results were overestimated, and the “true” effect when controlling for selective publication might be lower than the original pooled effect sizes. Note that we should be cautious to interpret the trim-and-fill results when the number of studies is small (i.e., k < 10).

### 4.3. Social Factors and Employee Performance and Well-Being

H2 tests the mediating effects of job crafting. First, we categorized our outcome variables as job performance and well-being. Table 3 showed the categorizations of work outcomes in which performance includes creativity, innovative work behavior, organizational citizenship behavior (OCB), positive work behavior, and task performance; and well-being includes affective commitment, job satisfaction, organizational identification, and thriving.

TSSEM was conducted to test the mediation effect. In the first stage, to calculate the pooled correlation matrix, we used 32 independent correlation matrix (N = 9156). First, we tested a fixed-effects model, the model fit indexes showed that χ2 (70) = 444.78, *p* < 0.01, comparative fit index (CFI) = 0.838, Tucker-Lewis index (TLI) = 0.831, and the root mean squared error of approximation (RMSEA) was 0.135, which was larger than 0.80, indicating bad fit. Therefore, in the second stage, we used a random-effects model to test our mediation hypothesis (Harrer, et al., 2019 [88]). The averaged correlation matrix based on the random-effects model was reported in Table 4. We found a medium-sized overall correlation between social factors and promotion-focused job crafting (r = 0.304, *p* < 0.01), and outcomes (performance and well-being) (r = 0.304, *p* < 0.001). Similarly, we found a positive association between promotion-focused job crafting and outcomes (r = 0.309, *p* < 0.001).

In stage 2, we used the pooled correlation matrix to fit the hypothesized structural model. The results were reported in Table 5 shows that promotion-focused job crafting positively mediated the relationship between social factors and well-being (b = 0.046, CI = [0.072, 0.103]), and promotion-focused job crafting positively mediated the relationship between social factors and performance (b = 0.054, CI = [0.081, 0.116]). Hence, H2 was supported (note that the significant mediation results cannot be interpreted as causality due to most of our included studies used a cross-sectional design).

However, due to the lack of data on the prevention-focused job crafting, we cannot test the mediating effect of prevention-focused job crafting. Therefore, Hypothesis 3 was not tested.

## 5. Discussion

This study aims to meta-analytically examine whether social factors related to employee job crafting, and how job crafting mediates the relationship between social factors and work outcomes (e.g., employee performance and well-being). Building on social capital theory, we categorized social factors as organizational insiders and outsiders. We found positive associations between social factors of organizational-insiders (e.g., constructive leadership and coworker support) and job crafting, whereas a nonsignificant relationship between social factors of organizational-outsider and job crafting (due to small sample sizes). Besides, leaderships are more important social factors than coworkers associated with employee job crafting. We further found that promotion-focused job crafting positively mediates the relationship between factors of organizational-insiders and work outcomes, whereas for prevention-focused job crafting we did not have enough studies to test our hypotheses. Our study suggests that social factors significantly shape employee job crafting behaviors and in particular promotion-focused job crafting can successfully transmit favorable social factors into improved work outcomes. Below, we explain our result of how specific social factors related to employee job crafting.

First, in this meta-analysis, we consider leadership as important social factors that associated with subordinates’ job crafting behavior. Among them, we found that empowering leadership and transformational leadership were two salient ones. From a regulatory-focus perspective, empowering and transformational leaderships are positively related to promotion-focused job crafting. From a resource-based perspective, empowering and transformational leaderships are positively related to seeking resources and seeking challenges (two forms of job crafting). These findings imply that when employees perceive their leaders are transformational and empowering, employees may have more social resources to enact their jobs and adjust work role boundaries and perceptions. Our results are in line with previous studies that transformational and empowering leaders are taken as supportive role models and vital job resources that foster employees to craft their jobs [19,20,41,44]. Contrarily, although it was not included in the meta-analytic analysis, we recognized from our included articles that destructive leaderships are negatively related to employee job crafting, such as abusive supervision [89] and paternalistic leadership/ authoritarianism [48]. In summary, our results demonstrate that supportive/favorable leaderships play an important role in driving employee (promotion-focused) job crafting, whereas destructive leaderships may have detrimental effects on employee (promotion-focused) job crafting.

Besides leadership, we found that coworkers’ support also related to employee’s job crafting and that coworkers’ job crafting influenced employee job crafting as well. These findings imply that coworkers are another important source that related to employees’ motivation to enact their jobs (although the pooled association with promotion job crafting was weaker than leadership). Hence, coworkers are also important social factors in terms of motivating and facilitating employee to adjust their work role boundaries and perceptions. As employees work in an increasingly interactive work environment and even need to collaborate with other coworkers in multidisciplinary teams [16,71,85], our results show that it is important for employees to learn from their coworkers and craft their jobs accordingly.

Finally, we found that promotion-focused job crafting positively mediates the relationship between social factors of organizational-insiders (e.g., leadership) and work outcomes, whereas prevention-focused job crafting did not. This finding implies that when employees work with supportive leaders or colleagues, an effective way to transform these social capitals into improved work performance and well-being is job crafting with a promotion focus (e.g., seeking resources, seeking challenges, and expansion-oriented task, relational, and cognitive crafting). We highlight that promotion-focused job crafting is an important behavioral mechanism through which employees can successfully accumulate vital job resources from leaders and colleagues, and in turn, resulting in beneficial consequences of their work performance and well-being [5].

### 5.1. Theoretical Implications

Our study contributes to the literature in three ways. First, we contribute to job crafting literature by integrating the social factors into the antecedents of job crafting. Although prior studies have recognized the individual factors (e.g., personality and personal resources) and job characteristics (e.g., autonomy and workload) that shape employee job crafting [4,7], our study advances this field by demonstrating how leadership, coworker, and organizational outsiders influence employee job crafting. This is important because the social elements of work play a critical role in shaping employees’ experiences and behaviors [11]. The interpersonal interactions and relationships are actually embedded in and influenced by the jobs, roles, and tasks that employees perform and enact [11]. Using a meta-analysis, our study systematically integrates job crafting model from a social/interpersonal perspective and delineates the antecedents (as well as outcomes) of job crafting based on a framework of organizational insiders and organizational outsiders. Therefore, our study adds to job crafting literature by expanding the antecedents of job crafting model.

Second, our study contributes to job design literature by showing that promotion-focused job crafting mediates the relationship between social factors and work outcomes. That is, promotion-focused job crafting (e.g., seeking resources, seeking challenges, and expansion-oriented task, relational, and cognitive crafting) is an effective self-initiated strategy that can successfully transform favorable social factors into improved work performance and well-being. We extend the work of Lichtenthaler and Fischbach [5] and uncover the roles of promotion-focused job crafting in the interpersonal context. By incorporating social learning and resource-based perspectives, this study underlines that job crafting is taken as an important social learning process; as well as a resource accumulation/conservation process by which employees can input social resources and output higher levels of their work performance and psychological well-being.

Third, our study contributes to leadership literature by identifying job crafting as an important mediator. Prior leadership literature has uncovered certain important intermediate mechanisms of how leadership related to employee outcomes [90]. For example, critical mediators, such as self-efficacy [91], trust in the leader [92], and identification with the leader [93] were positively linked the relationship between leadership and employee outcomes. However, relatively less is taken from a behavioral perspective to understand the leadership–employee outcomes relationship. By filling this gap, our study underscores the beneficial mediating role of employee job crafting in the relationship between leadership and employee outcomes. In this vein, we provide more nuanced insights on what specific actions and strategies employees can use to convert leaders’ attitudes, behaviors, and vision into their own work achievements.

### 5.2. Practical Implications

This study provides implications for management practice. First, our study indicates that supportive organizational insiders (in particular leadership) act as an important conduit for shaping employees’ proactive behaviors. Therefore, organizations should attempt to create a supportive work environment in order to cultivate employee job crafting behaviors. For example, organizations should hire and train leaders with more empowering and transformational abilities [94,95]. Besides, organizations should encourage colleagues’ helping, sharing, and mutual-supporting behaviors because these beneficial behaviors can be transmitted and learned among colleagues [71].

Second, our study suggests that promotion-focused job crafting serves as an effective mediator linking the relationship between organizational insiders and work outcomes. Therefore, organizations should facilitate such a bottom-up job redesign behavior. Managers should encourage employees to engage in promotion-focused job crafting [5]. For example, emerging studies demonstrate that job crafting training and interventions is an effective tool to enhance employee promotion-focused job crafting behaviors [96,97]. Hence, these job crafting based training programs could be introduced to increase employee performance and well-being. Specifically, managers could consider training employees how to seek resources (e.g., performance feedback, advice from coworkers, support from managers), which may help employees address their job demands [96] and have positive outcomes.

### 5.3. Limitations and Agenda for Future Research

In reviewing the literature on the antecedents of social factors of job crafting, we were impressed by how researchers have been taken to advance this field. But there are some important issues remain undressed (note that we only included published articles, this should be considered as a limitation, as the results might be biased by selective publication). Next, we provide a detailed research agenda for future study on job crafting on theoretical and methodology parts.

First, the focus of our meta-analysis was on the bivariate associations between social factors and job crafting as well as a mediating effect of promotion-focused job crafting. However, more complex relationships should be considered in future investigations. For example, although we uncovered the positive relationships between social factors and work outcomes through promotion-focused job crafting, another critical question is when such relationships could be amplified or constrained. In particular, individual factors such as promotion focus [18], organization-based self-esteem [98], and organization identification [19] have been found as moderators between social factors and job crafting. Future research needs to examine other boundary conditions of social factors on job crafting. In addition, prior studies have tended to investigate the moderators of the direct relationships between job crafting and various work outcomes [7,62]. We call for future studies to investigate how potential moderators influence the indirect relationships between social factors and work outcomes through job crafting. By doing so, the literature can capture a more comprehensive understanding of the different roles and mechanisms of employee job crafting.

Second, our meta-analyses only found the role of organizational insiders (e.g., leaders and colleagues) in employee job crafting and their work outcomes. Besides, these studies mostly focused on favorable/supportive leadership styles, for instance, empowering leadership, LMX, transformational leadership, ambidextrous leadership, charismatic leadership, servant leadership, and transactional leadership. However, employees sometimes may have to experience unfavorable leaderships such as paternalistic leadership and abusive leadership [20,48]. When employees work with these destructive leaders, it is important to understand how employees can react and respond to these leaders’ behaviors and proactively adapt their job boundaries. Therefore, future studies could investigate how and when unfavorable leaderships impact employee job crafting behaviors. By doing so, we can gain a more comprehensive understanding of whether job crafting a potent strategy when work environments become less favorable.

Besides examining the relationship between organizational-insiders and job crafting, future studies could pay more attention to the role of organizational-outsiders. For example, clients/customers factors and, family-based factors. Based on JD-R model, clients/customers factors can be seen as both social resources and stressors (i.e., demands) [26]. Clients/customers, on the one hand, provides employees with additional resources and motives to adjust job conditions; on the other hand, clients/customers’ overwhelming needs may exhaust employees’ energy and force employees to reduce such demands (i.e., prevention-focused job crafting). Therefore, clients/customers are important external factors but influence employees’ behaviors at work such as job crafting [99]. Future studies can dig into the relationship between clients/customers factors and employee job crafting. In addition to the external factors of clients/customers, family-based factors are also important for future studies to consider. The spill-over literature indicates that individuals’ behaviors and emotions are likely to spill over to another domain and influence individuals’ performance in another domain [60,71]. This literature also demonstrates that resources can be transferred within and across social and organizational contexts [55]. However, we only identified a small piece of studies examining the associations between job crafting and family factors (e.g., work-family conflicts and work-family enrichment) [51,71]. Unfortunately, those family factors were taken as the outcome of job crafting rather than the antecedent. This is a research gap that needs to be filled by future studies. To summarize, we call for future studies to empirically examine whether and how the factors of organizational outsiders (e.g., clients/customers, family-based factors) may shape employee job crafting behaviors during the work.

Third, we remind future studies to consider the cross-culture issues of employee job crafting. In our included studies, participants were from 17 different countries and areas, most of which were from western countries such as Germany, Netherlands, and United States. However, research suggests that individuals’ perceptions of leaders’ behaviors are very likely to be influenced by national culture and perform differently in different national settings [100,101]. For example, people in some cultures may accept a higher degree of unequally distributed power than do people in other cultures [102]. In a high-power distance culture, the relationship between leaders and subordinates is one of dependence; whereas in a low power distance society, the relationship between leaders and subordinates is one of interdependence [103]. Therefore, in some cultures with high power distance, employees may have less autonomy or have less motivation to change their job boundaries. For instance, in a recent meta-analysis, Li, Sun, Taris, Xing, and Peeters found that power distance moderates the relationship between leadership and work engagement [104]. Unfortunately, in our reviewed articles, no one has addressed the cross-culture issues. Thus, future studies can look into how national cultural characteristics moderate the relationship between social factors (e.g., organizational outsiders and insiders we mentioned in this study) and employee job crafting behaviors.

Finally, we draw attention to methodological issues for future study. In our reviewed articles, the majority were cross-sectional (43%) or multi-wave designs (49%). Moreover, 8% of studies used diaries design to capture the short-term dynamics of job crafting process within and between individuals in the work context. Nevertheless, no (laboratory or field) experimental studies have been conducted to investigate the causal effects of social factors on employee job crafting behaviors. Obviously, the cross-sectional, multi-wave, or diary design without dealing with the endogeneity bias issue, can only tell us the correlational relationships between these factors and job crafting behaviors, whereas no causal relationships were testified [105]. This is unfortunate, because the founded significant correlations could be caused by omitted variables or a situation in which social factors and job crafting could influence each other (i.e., endogeneity bias issue) [105]. Due to these methodology issues, the significant correlations and the indirect effects in our meta-analysis cannot be interpreted as causality. Therefore, to address this issue and test a causal effect of social factors on job crafting, we suggest that future studies can take three possible approaches: (a) using experimental designs (e.g., field-experiment or lab-experiment) to investigate how social factors influence employee job crafting; (b) using panel designs to investigate the potential reciprocal causal effects; (c) using instrumental regression model to reduce the endogeneity bias in survey designs (for technical issues, see [105,106,107]).

## 6. Conclusions

This study conducted a comprehensive meta-analysis of the relationships between social factors, job crafting, and work outcomes and integrated a general model of employee job crafting from a social/interpersonal perspective. We found that factors of organizational insiders were positively related to promotion-focused job crafting and promotion-focused job crafting plays a mediating role in the relationship between organizational insiders and work outcomes. This study contributes to job crafting literature by stressing the importance of the social factors in cultivating employee job crafting and the role that promotion-focused job crafting plays in transforming socially valuable resources into improved work outcomes. We suggest that it is important for organizations and management practitioners to be aware of the social factors of job crafting and to facilitate employee proactive behaviors in the form of promotion-focused job crafting.

## Figures and Tables

**Table 1:** Summary of meta-analytic relationships: social factors as correlates of job crafting (H1).

Variables	*k*	*N*	*r*	*ρ*	*SEρ*	Lower	Upper	*p*	Q	I^2^	H^2^	*z*Fisher	*ρ* _*_sensitivity analysis_*	*kTrim-and Fill*	*ρ_Trim-and Fill_*	*ρ* _*_measurement_*
Promotion focus job crafting																
Overall social factors	32	9263	0.332	0.361	0.039	0.292	0.426	<0.0001	404.067 ***	92.78%	13.840	0.378	0.341	32	0.361	0.416
Coworker support	3	519	0.231	0.237	0.068	0.108	0.358	0.0004	4.674 (0.09)	57.42%	2.350	0.242	no outlier	3	0.237	0.311
Leadership overall	22	6953	0.364	0.400	0.051	0.314	0.480	<0.0001	313.887 ***	94.15%	17.110	0.424	0.385	22	0.400	0.456
Empowering leadership	7	2262	0.320	0.338	0.031	0.282	0.391	<0.0001	12.673 *	47.58%	1.910	0.352	0.341	7	0.341	0.384
LMX	7	1791	0.264	0.277	0.062	0.161	0.385	<0.0001	38.024 ***	84.67%	6.520	0.285	0.213	10	0.174	0.320
Transformational leadership	5	1551	0.263	0.270	0.041	0.193	0.343	<0.0001	9.387 (0.05)	58.72%	2.420	0.276	no outlier	7	0.319	0.329
Servant leadership	3	1019	0.579	0.686	0.173	0.464	0.827	<0.0001	58.501 ***	96.34%	27.320	0.841	0.670	5	0.510	0.735
Prevention focus job crafting																
Overall social factors	9	2007	0.019	0.022	0.058	−0.091	0.134	0.7044	41.896 ***	83.94%	6.230	0.022	0.001	9	0.022	0.027

Note: *, *p* < 0.05; ***, *p* < 0.001; *k* = number of independent samples included; *ρ* = sample-size-weighted mean observed correlation; *SEρ* = standard error for population estimate; I^2^ is an index of heterogeneity computed as the percentage of variability in effects sizes that are due to true differences among the studies; Q provides information on whether there is statistically significant heterogeneity (i.e., yes or no heterogeneity). Overall social factors-two level-single *=* only include one effect size for each study; Overall social factors-two level-nested = for some studies included several effect sizes, which may not independent; *ρ* _*_sensitivity analysis_* = outlier removed sensitivity analyses; *kTrim-and fill* = number of independent samples included for trim-and-fill analysis; *ρ_Trim-and fill_* = trim-and-fil results; *ρ* _*_measurement =_* mean score correlation (corrected for unreliability for both variables and sampling error variance).

**Table 2:** Summary of meta-analytic relationship: social factors as correlates of specific job crafting behaviors (H1).

Variables	*k*	*N*	*r*	*ρ*	*SEρ*	Lower	Upper	*p*	Q	I^2^	H^2^	*z*Fisher	*ρ* _*_sensitivity analysis_*	*kTrim-and Fill*	*ρ_Trim-and Fill_*	*ρ* _*_measurement_*
Increasing challenge job demands																
Overall social factors	11	3195	0.201	0.209	0.037	0.138	0.277	<0.0001	35.737 ***	75.66%	4.11	0.212	0.186	12	0.186	0.255
empowering leadership	4	807	0.290	0.305	0.071	0.174	0.426	<0.0001	9.526 *	73.17%	3.73	0.316	no outliers	4	0.306	0.353
transformational leadership	3	1041	0.228	0.234	0.036	0.165	0.300	<0.0001	2.185 (0.34)	22.27%	1.29	0.238	no outliers	5	0.190	0.299
Increasing social job resources																
Overall social factors	10	3024	0.315	0.332	0.048	0.246	0.414	<0.0001	55.198 ***	84.95%	6.64	0.346	0.332	11	0.348	0.396
empowering leadership	4	807	0.343	0.368	0.104	0.181	0.530	0.0002	20.701 ***	87.40%	7.94	0.387	no outliers	4	0.369	0.432
transformational leadership	3	1055	0.348	0.367	0.096	0.196	0.517	<0.0001	13.852 **	88.39%	8.62	0.385	0367	3	0.367	0.451
Increasing structural job resources																
Overall social factors	6	2357	0.173	0.178	0.062	0.058	0.293	0.0039	44.879 ***	88.88%	8.99	0.180	0.178	6	0.178	0.215
transformational	3	1195	0.251	0.260	0.096	0.078	0.425	0.0056	18.799 ***	90.50%	10.52	0.266	0.260	3	0.260	0.312

Note: *, *p* < 0.05; **, *p* < 0.01; ***, *p* < 0.001; *k* = number of independent samples included; *ρ* = sample-size-weighted mean observed correlation; *SEρ* = standard error for population estimate; I^2^ is an index of heterogeneity computed as the percentage of variability in effects sizes that are due to true differences among the studies; Q provides information on whether there is statistically significant heterogeneity (i.e., yes or no heterogeneity). Overall social factors-two level-single *=* only include one effect size for each study; Overall social factors-two level-nested = for some studies included several effect sizes, which may not independent; *ρ* _*_sensitivity analysis_* = outlier removed sensitivity analyses; *kTrim-and fill* = number of independent samples included for trim-and-fill analysis; *ρ_Trim-and fill_* = trim-and-fil results; *ρ* _*_measurement =_* mean score correlation (corrected for unreliability for both variables and sampling error variance).

**Table 3:** Categorizations of work outcomes for Meta Structural Equation Modeling analysis.

Well-Being	Performance
Thriving	Creativity
Affective commitment	Innovative work behavior
Job satisfaction	Task performance
Organizational identification	Organizational citizenship behavior
	Positive work behavior

**Table 4:** Pooled correlation matrix based on the random-effects model.

Variables	1	2	3
1. Social factors	1		
2. Promotion-focused job crafting	0.304 ***	1	
3. Outcomes	0.304 ***	0.309 ***	1

*Note*: Outcomes include well-being and performance. *** *p* < 0.001.

**Table 5:** Parameter estimates and 95% confidence intervals for studies with different mediators (H2).

	Well-Being			Performance			Overall Outcomes		
Parameters	Estimate	Lower	Upper	Estimate	Lower	Upper	Estimate	Lower	Upper
b21	0.264	0.198	0.330	0.352	0.271	0.433	0.304	0.254	0.354
b32	0.272	0.181	0.362	0.231	0.154	0.306	0.239	0.182	0.296
b31	0.270	0.189	0.350	0.185	0.106	0.263	0.231	0.171	0.290
p22	0.930	0.891	0.961	0.876	0.812	0.927	0.907	0.875	0.935
p33	0.814	0.748	0.871	0.882	0.839	0.918	0.856	0.819	0.889
Indirect effect	0.046	0.072	0.103	0.054	0.081	0.116	0.054	0.073	0.094

Note: for well-being, *k* = 9, N = 3580; for performance, *k* = 14, N = 3532; overall outcomes, *k* = 31, N = 9156; b21 = social factors to promotion-focused job crafting; b31 = social factors to outcomes; b32 = job crafting to outcomes.

## Appendix A

((TS=“leadership” OR TS=“leader” OR TS=“supervisor” OR TS =“*leadership” OR TS=“leader support” OR TS=“supervisor support”) OR (TS=“ transformational leadership “ OR TS=“ charismatic leadership “ OR TS=“ charisma “ OR TS=“ vision “) OR (TS=“ authentic leadership “ OR TS=“ authentic leader “ OR TS=“ authenticity “ OR TS=“ authentic behavior “) OR (TS=“ ethical leadership “ OR TS=“ ethical leader “ OR TS=“ ethical integrity “ OR TS=“ ethical manager “ OR TS=“ moral manager “ OR TS=“ ethical climate “ OR TS=“ ethical context “ OR TS= “ moral leadership”) OR (TS=“ servant leadership “ OR TS=“ servant leader “ OR TS=“ servant organization “ OR TS=“ servant behavior “) OR (TS=“ empowering leadership “ OR TS=“ empowering leader “ OR TS=“ empowering manager “ OR TS=“ empowering behavior “) OR (TS=“ abusive supervision “ OR TS=“ abusive leader “ OR TS=“ abusive supervisor behavior “ OR TS=“ perceived abuse “ OR TS=“ perceived abusive supervision “) OR (TS=“ Paternalistic leadership” OR TS=“ authoritarianism “ OR TS=“ benevolence “ OR TS=“ moral leadership “) OR (TS=“ shared leadership “ OR TS=“ collective leadership “ OR TS=“ distributed leadership “ OR TS=“ team leadership “ OR TS=“ peer leadership “) OR (TS=“ Transactional leadership” OR TS=“ contingent reward “ OR TS=“ management by exception—active “ OR TS=“ management by exception—passive”) OR (TS=“colleague support” OR TS=“coworker support” OR TS=“colleague help” OR TS=“coworker help” OR TS=“colleague conflict” OR TS=“coworker conflict”) OR (TS=“work-family conflict” OR TS=“work-life conflict” OR TS=“work-nonwork conflict” OR TS=“work family interference” OR TS=“work-family facilitation” OR TS=“work-family enrichment” OR TS=“work-family spillover” OR TS=“work-family integration” OR TS=“family support”))

AND (TS=“Job crafting” OR TS=“active coping” OR TS=“crafting” OR TS=“seeking structural resources” OR TS=“seeking social resources” OR TS=“seeking challenges” OR TS=“ reducing demands” OR TS = “increasing structural job resources” OR TS = “decreasing hindering job demands” OR TS =“increasing social job resources” OR TS = “increasing challenging job demands”)

Note: TS = title, keywords, and abstract

## Appendix C

**Table A1:** A summary of selected studies in this meta-analysis.

Study ID	Authors	Year	Journal	Theoretical Framework: 1 = JD-R Model (Job Crafting Theory) 2 = Leadership Theory 3 = Other, Please Specify	Sample Size	Country Setting	Study Design 1 = Cross-Sectional; 2 = Lagged/Multi-Wave; 3 = Panel/Repeated Measure; 4 = Diary/ESM; 5 = Field-Experimental; 6 = Lab-Experimental;	Job Crafting Questionnaire 1 = Tims et al., 2013; 2 = Others, Specify
1	Afsar, Bilal and Masood, Mariam and Waheed Ali Umrani	2019	*Personnel Review*	1; 2; social exchange theory	325	Pakistan	1	1
2	Bakker, Arnold B and Rodriguez-Munoz, Alfredo and Vergel, Ana Isabel Sanz	2016	*Human Relations*	1; social cognitive theory	206	Poland, Romania, Lithuania, and The Netherlands	1	1
3	Bavik, Ali and Bavik, Yuen Lam and Tang, Pok Man	2017	*Cornell Hospitality Quarterly*	1; social learning theory	238	Macau, China	1	1
4	Domenico Berdicchia and Giovanni Masino	2019	*Journal of Management & Organization*	conservation of resources theory	162	Italy	1	1
5	Dash, Sanket Sunand and Vohra, Neharika	2019	*Management Research Review*	job characteristics model	624	India	1	1
6	Demerouti, Evangelia and Peeters, Maria C W	2018	*Journal of occupational and organizational psychology*	social contagion/impact theory	130	Netherlands	4	Petrou et al. (2012); Demerouti and Peeters, 2018
7	Diellza Gashi Tresi and Katarina Katja MiheliÄ	2018	*Personnel Review*	conservation of resources theory; LMX theory	204	Kosovo	1	1
8	Ding, He and Yu, Enhai and Chu, Xixi and Li, Yanbin and Amin, Kashif	2020	*Frontiers in Psychology*	1; social learning theory	260	China	2	Slemp and Vella-Brodrick (2013),
9	Esteves, T., & Pereira Lopes, M.	2017	*Western Journal of Nursing Research*	2	325	Portuguese	1	1
10	Goodall, SA	2018	master thesis	1; 2; conservation of resources theory	145	New Zealand	1	Nielsen and Simonsen Abildgaard, 2012
11	Guan Xiaoyu and Frenkel, Stephen	2018	*Chinese Management Studies*	1; job characteristics theory; conservation of resources theory; human resource management (HRM) process theory	455	China	2	1
12	Guan Xiaoyu and Frenkel, Stephen J	2019	*Human Relations*	1; conservation of resources theory; social exchange theory	406	China	2	1
13	Harju, LK and Schaufeli, WB and Hakanen, JJ	2018	*Journal of Managerial Psychology*	conservation of resources theory	237	unknown	2	1
14	Hetland, J., Hetland, H., Bakker, A. B., & Demerouti, E	2018	*European Management Journal*	1; transformational leadership theory	535	Norway	4	1
15	Holcombe, Kyla J	2017	PhD thesis	1; self-determination theory	120	United States	1	Dvorak, 2014
16	Kim, M and Beehr, TA and Kim, Minseo and Beehr, Terry A.	2018	*Journal of Leadership & Organizational Studies*	1; conservation of resources theory; person-job fit theory	325	United States	2	1
17	Kim, M and Beehr, TA and Kim, Minseo and Beehr, Terry A.	2020	*European Journal of Work and Organizational Psychology*	1; empowering leadership theory	276	United States	2	1
18	Kim, M and Beehr, TA and Kim, Minseo and Beehr, Terry A.	2019	*The International Journal of Human Resource Management*	1; conservation of resources theory	331	United States	2	Niessen et al. (2016).
19	Minseo Kim and Terry A. Beehr		Poster		276	United States	2	1
20	Kwon, N and Kim, M and Kim, MS	2019	*Sustainability*	affective event theory; leader member exchange theory	105	South Korea	4	1
21	Laurence, G. A	2010	PhD thesis	1	163	Japan and China	1	Hackman and Oldham, 1974; Sims, Szilagyi, and Keller, 1976
22	Lichtenthaler, PW and Fischbach, A	2018	*Leadership & Organization Development Journal*	1;	117	Germany	1	1
23	Loi, R and Xu, AJ and Chow, CWC and Chan, WWH	2020	*Journal of Occupational and Organizational Psychology*	1; conservation of resources theory	139	china	2	Slemp and Vella-Brodrick (2014)
24	Qian Lu	2018	conference proceeding	conservation of resources theory				
25	Luu, T and Le, V and Masli, E and Rajendran, D	2019	*Marketing Intelligence & Planning*	Social exchange theory	468	Vietnam	2	adapted from Tims et al.’s (2012) job crafting scale
26	Luu, TT and Dinh, K and Qian, D	2019	*European Business Review*	2; ambidextrous leadership	427	Vietnam	2	1
27	Luu, TT and Luu, Tuan Trong	2019	*Personnel Review*	conservation of resources theory	492	Vietnam	2	1
28	Luu, Tuan Trong	2020	*Industrial Marketing Management*	authentic leadership, conservation of resources theory	872	Vietnam	2	1
29	Ma, J., Zhou, X., Chen, R., & Dong, X.	2019	*International Journal of Hospitality Management*	2; ambidextrous leadership	290	China	2	1
30	Mancini, Victor S	2019	*Graduate Theses and Dissertations*	work-family balance	1411	United States	2	Slemp and Vella-Brodrick, 2013
31	Peeters, Maria CW and Arts, Richard and Demerouti, Evangelia	2016	*European Journal of Work and Organizational Psychology*	1; crossover theory	110		4	Petrou et al. (2012).
32	Qi, JP and Zhang, KY and Fu, XF and Zhao, XF and Wang, L	2019	*Social Behavior and Personality: an international journal*	2	212	China	1	Sekiguchi, Li, and Hosomi (2014)
33	Radstaak, M and Hennes, A	2017	*SA Journal of Industrial Psychology*	1,2	402	Netherland	1	1
34	Rastogi, Mansi and Chaudhary, Richa	2018	*Personnel Review*	1, work engagement	496	India	1	1
35	Riet, VD	2015	master thesis	2	171	Netherland	1	Petrou, Demerouti, Peeters, Schaufeli and Hetland (2012)
36	Tomoki Sekiguchi, Jie Li, and Masaki Hosomi	2017	*The Journal of Applied Behavioral Science*	1	564	Japan	1	Leana et al. (2009)
37	Deepa Sethi, Tanusree Chakraborty & Vikas Arya	2020	conference proceeding	missing	140	India	1	missing
38	Inyong Shin, Won-Moo Hur and Seongho Kang	2018	*International Journal of Environmental Research and Public Health*	1; person-job fit theory	175	South Korea	1	Slemp and Vella-Brodrick’s
39	Shin, Yuhyung and Hur, Won-Moo and Choi, Wook-Hee	2018	*The International Journal of Human Resource Management*	1	175	South Korean	2	Slemp and Vella-Brodrick’s (2014)
40	Solberg, Elizabeth and Wong, Sut I	2016	*The Leadership Quarterly*	leaders’ monitoring behaviors and work climate	143	Norway	2	Wrzesniewski, A., Bartel, A., and Wiesenfeld, B. (working paper)
41	Sylvi Thun and Arnold B. Bakker	2018	*Stress and Health*	1	331	Norway	1	1
42	Tuan, LTT and Luu Trong Tuan (Tuan Luu)	2018	*Public* Management *Review*	paternalistic leadership	527	Vietnam	2	1
43	van Gool, RJM	2019	PhD thesis	2	333	Netherland	1	1
44	Els Vanbelle	2017	PhD thesis	1; conservation of resources theory	583	Belgium	1	Vanbelle et al. (2016),
45	Wang, Hai-Jiang and Demerouti, Evangelia and Le Blanc, Pascale	2017	*Journal of Vocational Behavior*	1;2	115	Netherland	1	Petrou et al. (2012)
46	Wang, HB and Wang, XH and Li, JR	2018	*Asia Pacific Business Review*	LMX	289	China	2	Bizzi (2017)
47	Yang, Rui and Ming, Ying and Ma, Jianhong and Huo, Rongmian	2017	*Social Behavior and Personality*	servant leadership theory	544	China	1	Dvorak, (2014)
48	Zito, M., Colombo, L., Borgogni, L., Callea, A., Cenciotti, R., Ingusci, E., & Cortese, C. G	2019	*International Journal of Environmental Research and Public Health*	1	389	Italy	1	1
49	Philipp Wolfgang Lichtenthaler, Andrea Fischbach,	2018	*Leadership & Organization Development Journal*	1; self-regulatory theory	117	German	1	1 German version
50	Haijiang Wang dissertation chaper 4	2017	PhD thesis	Empower leadership	106	China	4	Petrou et al. (2012)
51	Haijiang Wang dissertation chaper 6	2017	PhD thesis	Empower leadership	231	China	2	Petrou et al. (2012)

## References

[1] Wrzesniewski A., Dutton J. (2001). Crafting a job: Revisioning employees as active crafters of their work. Acad. Manag. J..

[2] Bruning P.F., Campion M.A. (2018). A role-resource approach-avoidance model of job crafting: A multimethod integration and extension of job crafting theory. Acad. Manag. J..

[3] Oldham G.R., Fried Y. (2016). Job design research and theory: Past, present and future. Organ. Behav. Hum. Decis. Process..

[4] Rudolph C.W., Katz I.M., Lavigne K.N., Zacher H. (2017). Job crafting: A meta-analysis of relationships with individual differences, job characteristics, and work outcomes. J. Vocat. Behav..

[5] Lichtenthaler P.W., Fischbach A. (2019). A meta-analysis on promotion- and prevention-focused job crafting. Eur. J. Work Organ. Psychol..

[6] Tims M., Bakker A.B., Derks D., van Rhenen W. (2013). Job Crafting at the Team and Individual Level: Implications for Work Engagement and Performance. Group Organ. Manag..

[7] Zhang F., Parker S.K. (2019). Reorienting job crafting research: A hierarchical structure of job crafting concepts and integrative review. J. Organ. Behav..

[8] Bakker A.B., Tims M., Derks D. (2012). Proactive personality and job performance: The role of job crafting and work engagement. Hum. Relat..

[9] Kanten P. (2014). The antecedents of job crafting: Perceived organizational support, job characteristics and self-efficacy. Eur. J. Bus. Soc. Sci..

[10] Lee S., Shin Y., Baek S.I. (2017). The Impact of Job Demands. J. Appl. Bus. Res..

[11] Grant A.M., Parker S.K. (2009). 7 Redesigning Work Design Theories: The Rise of Relational and Proactive Perspectives. Acad. Manag. Ann..

[12] Seibert S.E., Kraimer M.L., Liden R.C. (2001). A Social Capital Theory of Career Success. Acad. Manag..

[13] Evans W.R., Carson C.M. (2005). A social capital explanation of the relationship between functional diversity and group performance. Team Perform. Manag..

[14] Perry-Smith J.E. (2006). Social Yet Creative: The Role of Social Relationships in Facilitating Individual Creativity L.. Acad. Manag. J..

[15] Lee J.Y., Lee Y. (2018). Job Crafting and Performance: Literature Review and Implications for Human Resource Development. Hum. Resour. Dev. Rev..

[16] Tims M., Parker S.K. (2020). How coworkers attribute, react to, and shape job crafting. Organ. Psychol. Rev..

[17] Afsar B., Masood M., Umrani W.A. (2019). The role of job crafting and knowledge sharing on the effect of transformational leadership on innovative work behavior. Pers. Rev..

[18] Hetland J., Hetland H., Bakker A.B., Demerouti E. (2018). Daily transformational leadership and employee job crafting: The role of promotion focus. Eur. Manag. J..

[19] Wang H.-J., Demerouti E., Le Blanc P. (2017). Transformational leadership, adaptability, and job crafting: The moderating role of organizational identification. J. Vocat. Behav..

[20] Esteves T., Pereira Lopes M. (2017). Leading to Crafting: The Relation Between Leadership Perception and Nurses’ Job Crafting. West. J. Nurs. Res..

[21] Loi R., Xu A.J., Chow C.W.C., Chan W.W.H. (2019). Linking customer participation to service employees’ work-to-family enrichment: The role of job crafting and OBSE. J. Occup. Organ. Psychol..

[22] Mirjam R., Ayla H. (2017). Leader—Member exchange fosters work engagement: The mediating role of job crafting. SA J. Ind. Psychol..

[23] Tims M., Bakker A.B., Derks D. (2013). The impact of job crafting on job demands, job resources, and well-being. J. Occup. Health Psychol..

[24] Wrzesniewski A., Lobuglio N., Dutton J.E., Berg J.M. (2013). Job crafting and cultivating positive meaning and identity in work. Advances in Positive Organizational Psychology.

[25] Tims M., Bakker A.B. (2010). Job crafting: Towards a new model of individual job redesign. SA J. Ind. Psychol..

[26] Bakker A.B., Demerouti E. (2017). Job demands-resources theory: Taking stock and looking forward. J. Occup. Health Psychol..

[27] Tims M., Bakker A.B., Derks D. (2012). Development and validation of the job crafting scale. J. Vocat. Behav..

[28] Bindl U.K., Unsworth K.L., Gibson C.B., Stride C.B. (2019). Job crafting revisited: Implications of an extended framework for active changes at work. J. Appl. Psychol..

[29] Tory Higgins E. (1997). Beyond pleasure and pain. Am. Psychol..

[30] Demerouti E., Bakker A.B., Halbesleben J.R.B. (2015). Productive and counterproductive job crafting: A daily diary study. J. Occup. Health Psychol..

[31] Rudolph C.W., Chang K., Rauvola R.S., Zache H. (2020). Meta-analysis in vocational behavior: A systematic review and recommendations for best practices. J. Vocat. Behav..

[32] Wu C.H., Li W.D. (2016). Individual differences in proactivity: A developmental perspective. Proactivity at Work: Making Things Happen in Organizations.

[33] Parker S.K., Bindl U.K., Strauss K. (2010). Making things happen: A model of proactive motivation. J. Manag..

[34] Berdicchia D., Nicolli F., Masino G. (2016). Job enlargement, job crafting and the moderating role of self-competence. J. Manag. Psychol..

[35] Kim H., Im J., Qu H. (2018). Exploring antecedents and consequences of job crafting. Int. J. Hosp. Manag..

[36] Wang H., Demerouti E., Bakker A.B. (2016). A review of job crafting research: The role of leader behaviors in cultivating successful job crafters. Proactivity Work Mak. Things Happen Organ..

[37] Fang R., Duffy M.K., Shaw J.D. (2011). The organizational socialization process: Review and development of a social capital model. J. Manag..

[38] Miller V.D., Jablin F.M. (1991). Information Seeking During Organizational Entry: Influences, Tactics, and a Model of the Process. Acad. Manag. Rev..

[39] Bavik A., Bavik Y.L., Tang P.M. (2017). Servant Leadership, Employee Job Crafting, and Citizenship Behaviors: A Cross-Level Investigation. Cornell Hosp. Q..

[40] Ding H., Yu E., Chu X., Li Y., Amin K. (2020). Humble Leadership Affects Organizational Citizenship Behavior: The Sequential Mediating Effect of Strengths Use and Job Crafting. Front. Psychol..

[41] Kim M., Beehr T.A. (2018). Can Empowering Leaders Affect Subordinates’ Well-Being and Careers Because They Encourage Subordinates’ Job Crafting Behaviors?. J. Leadersh. Organ. Stud..

[42] Kim M., Beehr T.A. (2020). Job crafting mediates how empowering leadership and employees’ core self-evaluations predict favourable and unfavourable outcomes. Eur. J. Work Organ. Psychol..

[43] Lichtenthaler P.W., Fischbach A. (2018). Leadership, job crafting, and employee health and performance. Leadersh. Organ. Dev. J..

[44] Thun S., Bakker A.B. (2018). Empowering leadership and job crafting: The role of employee optimism. Stress Health.

[45] Harju L.K., Schaufeli W.B., Hakanen J.J. (2018). A multilevel study on servant leadership, job boredom and job crafting. J. Manag. Psychol..

[46] Einarsen S., Aasland M.S., Skogstad A. (2007). Destructive leadership behaviour: A definition and conceptual model. Leadersh. Q..

[47] Lavoie-Tremblay M., Fernet C., Lavigne G.L., Austin S. (2016). Transformational and abusive leadership practices: Impacts on novice nurses, quality of care and intention to leave. J. Adv. Nurs..

[48] Tuan L.T. (2018). Behind the influence of job crafting on citizen value co-creation with the public organization: Joint effects of paternalistic leadership and public service motivation. Public Manag. Rev..

[49] Hung T.K., Wang C.H., Tian M., Yang Y.J. (2020). A cross-level investigation of team-member exchange on team and individual job crafting with the moderating effect of regulatory focus. Int. J. Environ. Res. Public Health.

[50] Shin I., Hur W.M., Kang S. (2018). How and when are job crafters engaged at work?. Int. J. Environ. Res. Public Health.

[51] Demerouti E., Peeters M.C.W. (2018). Transmission of reduction-oriented crafting among colleagues: A diary study on the moderating role of working conditions. J. Occup. Organ. Psychol..

[52] Bizzi L. (2017). Network characteristics: When an individual’s job crafting depends on the jobs of others. Hum. Relat..

[53] Halbesleben J.R.B., Buckley M.R. (2004). Managing customers as employees of the firm: New challenges for human resources management. Pers. Rev..

[54] Auh S., Bell S.J., McLeod C.S., Shih E. (2007). Co-production and customer loyalty in financial services. J. Retail..

[55] Hobfoll S.E., Halbesleben J., Neveu J.P., Westman M. (2018). Conservation of resources in the organizational context: The reality of resources and their consequences. Annu. Rev. Organ. Psychol. Organ. Behav..

[56] Chen S., Westman M., Hobfoll S.E. (2015). The commerce and crossover of resources: Resource conservation in the service of resilience. Stress Health.

[57] Hobfoll S.E. (2002). Social and Psychological Resources and Adaptation. Rev. Gen. Psychol..

[58] Aron A., Aron E.N., Norman C. (2007). Self-expansion Model of Motivation and Cognition in Close Relationships and Beyond. Blackwell Handb. Soc. Psychol. Interpers. Process..

[59] Wayne J.H., Randel A.E., Stevens J. (2006). The role of identity and work-family support in work-family enrichment and its work-related consequences. J. Vocat. Behav..

[60] Greenhaus J.H., Powell G.N., Greenhaus J.H., Powell G.N. (2006). When Work and Family Are Allies: A Theory of Work-Family Enrichment. Acad. Manag. Rev..

[61] King L.A., Mattimore L.K., King D.W., Adams G.A., Journal S., May N., King L. (1995). A Family Support Inventory for Workers: A New Measure of Perceived Social Support from Family Members. J. Organ. Behav..

[62] Cheng J.C., Yang O.-Y. (2018). Hotel employee job crafting, burnout, and satisfaction: The moderating role of perceived organizational support. Int. J. Hosp. Manag..

[63] Lavassani K.M., Movahedi B. (2014). Developments in Theories and Measures of Work-Family Relationships: From conflict to balance. Contemp. Res. Organ. Manag. Adm..

[64] Yavas U., Babakus E., Karatepe O.M. (2008). Attitudinal and behavioral consequences of work-family conflict and family-work conflict: Does gender matter?. Int. J. Serv. Ind. Manag..

[65] Demerouti E., Cropanzano R. (2017). The buffering role of sportsmanship on the effects of daily negative events. Eur. J. Work Organ. Psychol..

[66] Petrou P., Demerouti E., Peeters M.C.W., Schaufeli W.B., Hetland J. (2012). Crafting a job on a daily basis: Contextual correlates and the link to work engagement. J. Organ. Behav..

[67] Demerouti E., Bakker A.B., Gevers J.M.P. (2015). Job crafting and extra-role behavior: The role of work engagement and flourishing. J. Vocat. Behav..

[68] Slemp G.R., Vella-Brodrick D.A. (2014). Optimising Employee Mental Health: The Relationship Between Intrinsic Need Satisfaction, Job Crafting, and Employee Well-Being. J. Happiness Stud..

[69] Slemp G.R., Kern M.L., Vella-Brodrick D.A. (2015). Workplace Well-Being: The Role of Job Crafting and Autonomy Support. Psychol. Well Being.

[70] Akkermans J., Tims M. (2017). Crafting your Career: How Career Competencies Relate to Career Success via Job Crafting. Appl. Psychol..

[71] Peeters M.C.W., Arts R., Demerouti E. (2016). The crossover of job crafting between coworkers and its relationship with adaptivity. Eur. J. Work Organ. Psychol..

[72] Guan X., Frenkel S. (2018). How HR practice, work engagement and job crafting influence employee performance. Chin. Manag. Stud..

[73] Zito M., Colombo L., Borgogni L., Callea A., Cenciotti R., Ingusci E., Cortese C.G. (2019). The nature of job crafting: Positive and negative relations with job satisfaction and work-family conflict. Int. J. Environ. Res. Public Health.

[74] Harari M.B., Parola H.R., Hartwell C.J., Riegelman A. (2020). Literature searches in systematic reviews and meta-analyses: A review, evaluation, and recommendations Michael. J. Vocat. Behav..

[75] Siddaway A.P., Wood A.M., Hedges L.V. (2019). How to Do a Systematic Review: A Best Practice Guide for Conducting and Reporting Narrative Reviews, Meta-Analyses, and Meta-Syntheses. Annu. Rev. Psychol..

[76] Shin Y., Hur W.M., Choi W.H. (2020). Coworker support as a double-edged sword: A moderated mediation model of job crafting, work engagement, and job performance. Int. J. Hum. Resour. Manag..

[77] Luu T.T. (2019). Green human resource practices and organizational citizenship behavior for the environment: The roles of collective green crafting and environmentally specific servant leadership. J. Sustain. Tour..

[78] Tuan L.T. (2019). Effects of environmentally-specific servant leadership on green performance via green climate and green crafting. Asia Pacific J. Manag..

[79] Hunter J.E., Schmidt F.L., Le H. (2006). Implications of direct and indirect range restriction for meta-analysis methods and findings. J. Appl. Psychol..

[80] Viechtbauer W. (2010). Conducting meta-analyses in R with the metafor. J. Stat. Softw..

[81] Assink M., Wibbelink C.J.M. (2016). Fitting three-level meta-analytic models in R: A step-by-step tutorial. Quant. Methods Psychol..

[82] Van den Noortgate W., López-López J.A., Marín-Martínez F., Sánchez-Meca J. (2014). Meta-analysis of multiple outcomes: A multilevel approach. Behav. Res. Methods.

[83] Borenstein M., Hedges L.V., Rothstein H.R. (2011). Introduction to Meta-Analysis.

[84] Cheung M.W.L. (2014). Modeling dependent effect sizes with three-level meta-analyses: A structural equation modeling approach. Psychol. Methods.

[85] Bakker A.B., Rodríguez-Muñoz A., Sanz Vergel A.I. (2016). Modelling job crafting behaviours: Implications for work engagement. Hum. Relat..

[86] Laurence G.A. (2010). Workaholism and Expansion and Contraction Oriented Job Crafting: The Moderating Effects of Individual and Contextual Factors.

[87] Rastogi M., Chaudhary R. (2018). Job crafting and work-family enrichment: The role of positive intrinsic work engagement. Pers. Rev..

[88] Harrer M., Adam S.H., Baumeister H., Cuijpers P., Karyotaki E., Auerbach R.P., Kessler R.C., Bruffaerts R., Berking M., Ebert D.D. (2019). Internet interventions for mental health in university students: A systematic review and meta-analysis. Int. J. Methods Psychiatr. Res..

[89] Luu T.T. (2019). Discretionary HR practices and employee well-being: The roles of job crafting and abusive supervision. Pers. Rev..

[90] Inceoglu I., Thomas G., Chu C., Plans D., Gerbasi A. (2018). Leadership behavior and employee well-being: An integrated review and a future research agenda. Leadersh. Q..

[91] Hentrich S., Zimber A., Garbade S., Nienhaus A., Petermann F. (2017). Relationships Between Transformational Leadership and Health: The Mediating Role of Perceived Job Demands and Occupational Self-Efficacy. Int. J. Stress Manag..

[92] Braun S., Peus C., Weisweiler S., Frey D. (2013). Transformational leadership, job satisfaction, and team performance: A multilevel mediation model of trust. Leadersh. Q..

[93] Lian H., Brown D.J., Tanzer N.K., Che H. (2011). Distal charismatic leadership and follower effects: An examination of Conger and Kanungo’s conceptualization of charisma in China. Leadership.

[94] Arthur C.A., Hardy L. (2014). Transformational leadership: A quasi-experimental study. Leadersh. Organ. Dev. J..

[95] Martin S.L., Campbell E.M. (2013). Directive versus empowering leadership: A field experiment comparing impacts on task proficiency and proactivity. Acad. Manag. J..

[96] Van den Heuvel M., Demerouti E., Peeters M.C.W. (2015). The job crafting intervention: Effects on job resources, self-efficacy, and affective well-being. J. Occup. Organ. Psychol..

[97] Van Wingerden J., Derks D., Bakker A.B. (2017). The Impact of Personal Resources and Job Crafting Interventions on Work Engagement and Performance. Hum. Resour. Manag..

[98] Fu X., Zhao X., Qi J., Zhang K., Wang L. (2019). The effects of leader–member exchange, internal social capital, and thriving on job crafting. Soc. Behav. Pers..

[99] Dormann C., Zapf D. (2004). Customer-Related Social Stressors and Burnout. J. Occup. Health Psychol..

[100] Adler N.J. (1983). Cross-cultural management research: The ostrich and the trend. Acad. Manag. Rev..

[101] Hofstede G., Bond M.H. (1988). The Confucius Connection: From Cultural Roots to Economic Growth. Organ. Dyn..

[102] Smith A., Hume E.C. (2005). Linking culture and ethics: A comparison of accountants’ ethical belief systems in the individualism/collectivism and power distance contexts. J. Bus. Ethics.

[103] Schermerhorn J.R., Bond M.H. (1997). Cross-cultural leadership dynamics in collectivism and high power distance settings. Leadersh. Organ. Dev. J..

[104] Li P., Sun J., Taris T.W., Xing L., Peeters M.C.W. (2020). Country differences in the relationship between leadership and employee engagement: A meta-analysis. Leadersh. Q..

[105] Antonakis J., Bendahan S., Jacquart P., Lalive R. (2010). On making causal claims: A review and recommendations. Leadersh. Q..

[106] Daryanto A. (2020). EndoS: An SPSS macro to assess endogeneity. Quant. Methods Psychol..

[107] Maydeu-Olivares A., Shi D., Fairchild A.J. (2020). Estimating causal effects in linear regression models with observational data: The instrumental variables regression model. Psychol. Methods.

